# From data to decisions: a paradigm shift in fruit agriculture through the integration of multi-omics, modern phenotyping, and cutting-edge bioinformatic tools

**DOI:** 10.3389/fpls.2025.1707289

**Published:** 2025-12-10

**Authors:** Patricia Pacheco-Ruiz, Sonia Osorio, José G. Vallarino

**Affiliations:** Instituto de Hortofruticultura Subtropical y Mediterránea "La Mayora", Universidad de Málaga-Consejo Superior de Investigaciones Científicas (IHSM-UMA-CSIC), Málaga, Spain

**Keywords:** fruit breeding, multi-omics integration, high-throughput phenotyping, machine learning, genomic selection, CRISPR, genetic diversity, agricultural biotechnology

## Abstract

Fruit agriculture is undergoing a profound transformation driven by multi-omics, high-throughput phenotyping, and machine learning–driven bioinformatics. However, we demonstrate that this technological revolution has paradoxically created a ‘valley of death’ where most of genomic discoveries fail to reach farmers’ fields. While we can now identify beneficial alleles in days and edit genomes in weeks, it still takes 10 years and 14,5 million euros to deliver a single improved cultivar to European markets - the same timeline as 30 years ago. This review exposes how data abundance has shifted, not eliminated, the fundamental bottlenecks in fruit crop improvement. We critically assess how these tools reshape genetic and metabolic diversity, emphasizing both their transformative promises and structural limitations. We highlight three persistent gaps: the challenge of integrating heterogeneous multi-omics datasets, the phenotyping bottleneck for complex traits, and the tension between innovation and biodiversity conservation. By framing fruit breeding as a “data-to-decisions” challenge, we outline the systemic changes needed for sustainable, resilient, and high-quality fruit production.

## 1 Introduction

A rapidly expanding global population, projected to reach nearly 10 billion by 2050 (Ghosh et al., 2024), will drive a 35–56% increase in global food demand, which critically endangers food security (van van Dijk et al., 2021). This demographic pressure is compounded by the increasingly severe impacts of climate change, including unpredictable weather patterns, heat stress, water scarcity, and the proliferation of new pests and diseases (Bhattacharjee et al., 2022). Simultaneously, evolving consumer preferences are placing a greater emphasis on the nutritional quality, flavor, and sustainability of commercial fruits (Dobson and Marangoni, 2023; Harper et al., 2022; Paakki et al., 2022), introducing an additional layer of complexity to the field.

Nowadays, the fruit industry constitutes a massive global enterprise, with its market value surpassing $600 billion in 2023, representing over 50% growth since 2010 (FAOSTAT, 2025). This remarkable growth is attributed to the fact that fruits serve as critical sources of essential micronutrients and a vast array of phytochemicals (such as antioxidants, polyphenols, flavonoids, and phytosterols) that are not found in such diverse abundance in other foods (Marcillo-Parra et al., 2021; Sayago-Ayerdi et al., 2021; Whitehead et al., 2022). These compounds are widely recognized for their protective effects against multiple diseases such as cancer, cardiovascular disorders, diabetes and neurodegenerative conditions (Bazzano et al., 2002; Essa et al., 2023; Farvid et al., 2021; Franco et al., 2023; Zhou et al., 2021). Furthermore, beyond direct consumption, fruits and their industrial by-products are highly valuable raw materials for other industries, including food and beverage production (Anantachoke et al., 2023; Germerdonk et al., 2025), cosmetics (García-González et al., 2025; Osorio et al., 2021; Sharafan et al., 2023) and pharmaceuticals (Hussain et al., 2023; Ong and Nyam, 2022; Zhao et al., 2024), presenting them as sustainable and/or vegan alternatives to conventional products, further increasing their economic and social worth.

However, the remarkable success of modern fruit varieties in achieving high yields and uniform appearance has come at considerable cost (Clement et al., 2010; Fuller and Stevens, 2019; Wang et al., 2023). Intensive selection has narrowed genetic diversity, diluted flavor and nutritional value, and increased vulnerability to pests and climate stress, which now threaten both sustainability and food security (Bhardwaj et al., 2024; Brown and Hodgkin, 2015; Clement et al., 2010). Heavy dependence on pesticides and monocultures further amplifies ecological risks, while consumer dissatisfaction with flavor and quality has fueled demand for local, niche markets (Jacobsen et al., 2013; Klee and Tieman, 2018). These trends expose structural weaknesses in industrial fruit production and highlight an urgent need for a paradigm shift in breeding strategies; one that integrates yield, resilience, and sensory quality within a biodiversity-centered and sustainable framework.

Genetic diversity lies at the foundation of fruit crop improvement, providing the raw material upon which natural and artificial selection act (Swarup et al., 2021). This diversity is indispensable for fruit breeding programs, as it enables the development of better quality and resilient cultivars (Abbas et al., 2023; D’Amelia et al., 2021; Francini et al., 2020). It arises from variations in gene sequences, regulatory elements, copy number, and structural rearrangements, all of which influence gene expression and metabolic profiles (Bosman et al., 2023; Gu et al., 2016; Wang N. et al., 2024). The dynamic relationship between genomic and metabolic diversity is further modulated by genotype-by-environment (GxE) interactions, where environmental factors reconfigure biochemical pathways, resulting in significant phenotypic outcomes (Ali et al., 2021; Espley and Jaakola, 2023; Medda et al., 2022; Muños et al., 2011; Song et al., 2024). Numerous studies have investigated the genetic diversity present in major fruit crops. For example, in persimmon—an important exotic crop—a large-scale evaluation of 242 accessions using morphological and SSR markers revealed substantial genetic variation within the collection, with 28 out of 66 studied traits exhibiting a coefficient of variation (CV) greater than 40% (Li Y. et al., 2023). Conversely, other studies have focused on assessing the loss of genetic diversity in these crops to evaluate the extent of genetic erosion resulting from past breeding strategies. Gil-Ariza and colleagues reported a reduction of around 35% in the genetic diversity of cultivated strawberries due to the domestication process (Gil-Ariza et al., 2009). This observation is consistent with findings by Aharoni and colleagues, who compared the genetic backgrounds of wild strawberry (*Fragaria vesca*) and cultivated strawberry (*Fragaria × ananassa*) using cDNA microarray analysis. They identified a loss of expression in a terpene synthase gene responsible for the synthesis of a key aroma compound unique to wild cultivars, highlighting the impact of domestication on genetic and metabolic diversity (Aharoni et al., 2004).

Fruit breeding faces unique biological constraints beyond genetic diversity loss. Long generation times—often spanning 3 (peach) to 15 years (avocado) (van Nocker and Gardiner, 2014)—make QTL validation through backcrossing prohibitively slow and highlight the need for highly accurate genomic prediction methods. The high heterozygosity and outcrossing nature of many fruit crops result in each individual being genetically unique, necessitating genomic models that account for dominance and epistatic effects to improve prediction accuracy (Ferrão et al., 2021; Shen et al., 2022; Zingaretti et al., 2020). Low linkage disequilibrium (LD) in outcrossing populations further reduces the power of GWAS unless extremely high SNP densities are employed, complicating the identification of trait-associated loci (Hu et al., 2025; Nimmakayala et al., 2016). Clonality and vegetative propagation enable repeated evaluation of the same genotype, but these practices introduce complexities in experimental design, particularly for assessing genotype-by-environment (GxE) interactions (Gonçalves et al., 2020). Additionally, the widespread use of grafting in fruit trees introduces rootstock-scion interactions, which involve complex physiological, molecular, and epigenetic mechanisms that must be considered as additional effects in genomic models to accurately predict fruit quality and stress responses (Mauro et al., 2022; Wang et al., 2025). Addressing these biological and methodological challenges is essential for advancing omic-assisted breeding, especially in perennial fruit crops.

Additionally, other nonspecific limitations sum up to the challenge. Plant breeding has evolved from the serendipitous selections of early farmers to systematic hybridization based on Mendelian genetics. Later, it evolved to the molecular breeding strategies of the genomic era, where DNA markers and genetic engineering accelerated the development of elite fruit crops (Ahmadzai et al., 2024; Mansoor and Kim, 2024). Yet, these methods were often applied in isolation, limiting their effectiveness and complicating translation into real-world agricultural contexts. Today, the field has entered the post-genomic era, characterized by high-throughput, multi-omic integration that combines genomics, metabolomics, phenomics and advanced bioinformatics to provide a more holistic view of trait architecture (Adhikari et al., 2023).

On one side, high-throughput sequencing technologies now enable the rapid and cost-effective generation of vast genomic datasets, making comprehensive genomic studies more accessible to researchers worldwide and providing a foundational blueprint for breeding efforts (Shimizu et al., 2020; Tiwari et al., 2022). High-throughput metabolomics have accelerated the screening process of thousands of fruits, allowing the simultaneous analysis of many traits, increasing the statistical power of the analysis, and serving as a critical bridge between the genetic background (genotype) and the observable traits (phenotype) (Campa et al., 2024; Mansoor and Kim, 2024; Singh et al., 2025). Similarly, high-throughput phenotyping has revolutionized the phenotyping process by employing non-invasive technologies that collect data rapidly and precisely (Fu and Jiang, 2022; Angidi et al., 2025). For example, spectral imaging, including Visible and Near-Infrared (Vis-NIR) spectroscopy, can accurately and quickly measure traits such as fruit size, shape, color, and firmness (Zhao et al., 2024). However, the true power of integrating multi-omics and modern phenotyping lies in the ability to interpret the resulting deluge of data and their practical application to real world scenarios. Advanced computational techniques, including supervised and unsupervised machine learning algorithms and Artificial Intelligence (AI) models, are now essential for identifying meaningful patterns, building predictive models, and ultimately enabling the selection of superior cultivars for breeding programs (Ferrão et al., 2023; Gerakari et al., 2025). AI models can analyze integrated genomic and environmental data to predict fruit quality and resilience, helping breeders select the most promising lines for a given climate or market. This capacity to transform complex data into actionable insights forms the foundation of the actual breeding paradigm.

This holistic approach, as demonstrated in recent studies on strawberry fruit (*Fragaria × ananassa*) for flavor dissection (Fan et al., 2022) and peach fruit (*Prunus persica* (L.) *Batsch*) for investigating post-harvest ripening (Sirangelo et al., 2022), allows for a more comprehensive understanding of G×E interactions and their effects on final fruit quality and resilience (Marais et al., 2013; Marsh et al., 2021; Xu et al., 2022). By evaluating elite germplasm and novel breeding lines across diverse environmental conditions, researchers can generate data that more accurately reflects real-world performance, effectively bridging the gap between laboratory discoveries and the multifaceted demands of modern agriculture (Adunola et al., 2024; Chavarría-Perez et al., 2020; Willman et al., 2022).

Yet despite these technological breakthroughs, a critical disconnect persists between laboratory achievements and field implementation—what we term the ‘Agri-Tech Valley of Death.’ Borrowed from pharmaceutical and biotechnology sectors where it describes the gap between basic research and commercial application (Neumann et al., 2018; Smyth et al., 2014), this concept in fruit breeding encompasses the systematic failures that prevent genomic discoveries from translating into cultivars that farmers plant and consumers purchase. Unlike the linear progression from gene discovery to variety release depicted in scientific literature, the reality is a complex landscape of biological uncertainties, regulatory mazes, economic barriers, and phenotypic complexities that can extend the journey from laboratory to orchard by decades—if the journey is completed at all (Alvarez et al., 2021).

During the period from 1972 to 1998 in Dresden-Pillnitz, Germany, an apple breeding program aimed at developing scab-resistant cultivars commenced with approximately 52,000 seeds. However, only three apple cultivars were ultimately released to the market. This limitation was further exemplified in 2004 when scab-resistant cultivars constituted less than 5-6% of the German apple market, highlighting a significant gap between biotechnological advances and their practical commercial application (Kole, 2011). Moreover, there exists a notable deficiency in the quantification of the success rates of modern biotechnological interventions and their tangible implementation in commercial breeding programs. Accurate quantification of these success metrics in fruit crop improvement would be instrumental in elucidating existing barriers and in guiding the development of innovative strategies to address them. This phenomenon is not merely a matter of temporal delay but represents a profound structural challenge in which sophisticated technical solutions, often developed under controlled experimental conditions, fail to translate effectively into the complex biological, social, and economic realities of agricultural systems.

In fruit crop improvement, this valley of death manifests through three interconnected bottlenecks that no amount of data or computational power can bypass. First, the biological-regulatory bottleneck: even precise genetic modifications must navigate years of backcrossing, multi-environment trials, and regulatory frameworks designed for twentieth-century technologies. Second, the socioeconomic bottleneck: the concentration of advanced breeding technologies in well-funded institutions creates a widening gap between those who generate genomic insights and the farmers who need resilient varieties. Third, the ‘last mile’ phenotypic bottleneck: our inability to efficiently measure and predict the complex, emergent traits that determine market success—flavor that persists through cold storage, texture that survives transport, aroma that develops post-harvest. These bottlenecks ensure that while we celebrate sequencing breakthroughs and machine learning models, farmers continue planting decades-old varieties and consumers continue lamenting the loss of fruit quality.

Tropical fruit breeding is one of the areas affected by this valley of death. It is not only limited by the extended juvenile phases, high heterozygosity and inadequate data standardization, but also for the disconnection between institutions and industries. Kholová et al. (2024) discussed these challenges in low-income agricultural systems and reported the rate of adoption of new cultivars in sub-Saharan Africa remain below 35%, reflecting fundamental misalignment between breeding priorities and farmer’s needs (Kholová et al., 2024). To address these systemic challenges, the authors proposed integrated solutions, such as participatory frameworks that build trust through co-creation with end-users and strategic investment rebalanced from supply-side innovation toward addressing the behavioral and contextual barriers that prevent field deployment.

However, even with growing recognition of these social and institutional challenges, similar obstacles persist on the technological front. The promise of integrating multi-omics data for real-world applications remains a tantalizing, yet unfulfilled, prospect. The seamless transition from sophisticated data analysis to tangible solutions is consistently thwarted by persistent and often overlooked challenges. This work provides a holistic synthesis of omics, phenotyping, and computational approaches, with a special emphasis on their integration for fruit crops and their impacts on fruit genetic diversity. We discuss the limitations of past breeding strategies and how the new approaches respond to them, exploring their potential within the field, the current gaps and the future directions of fruit breeding strategies under the current global scenario.

## 2 From traditional breeding to modern approaches: shaping genomic and metabolomic diversity

**Figure 1:**
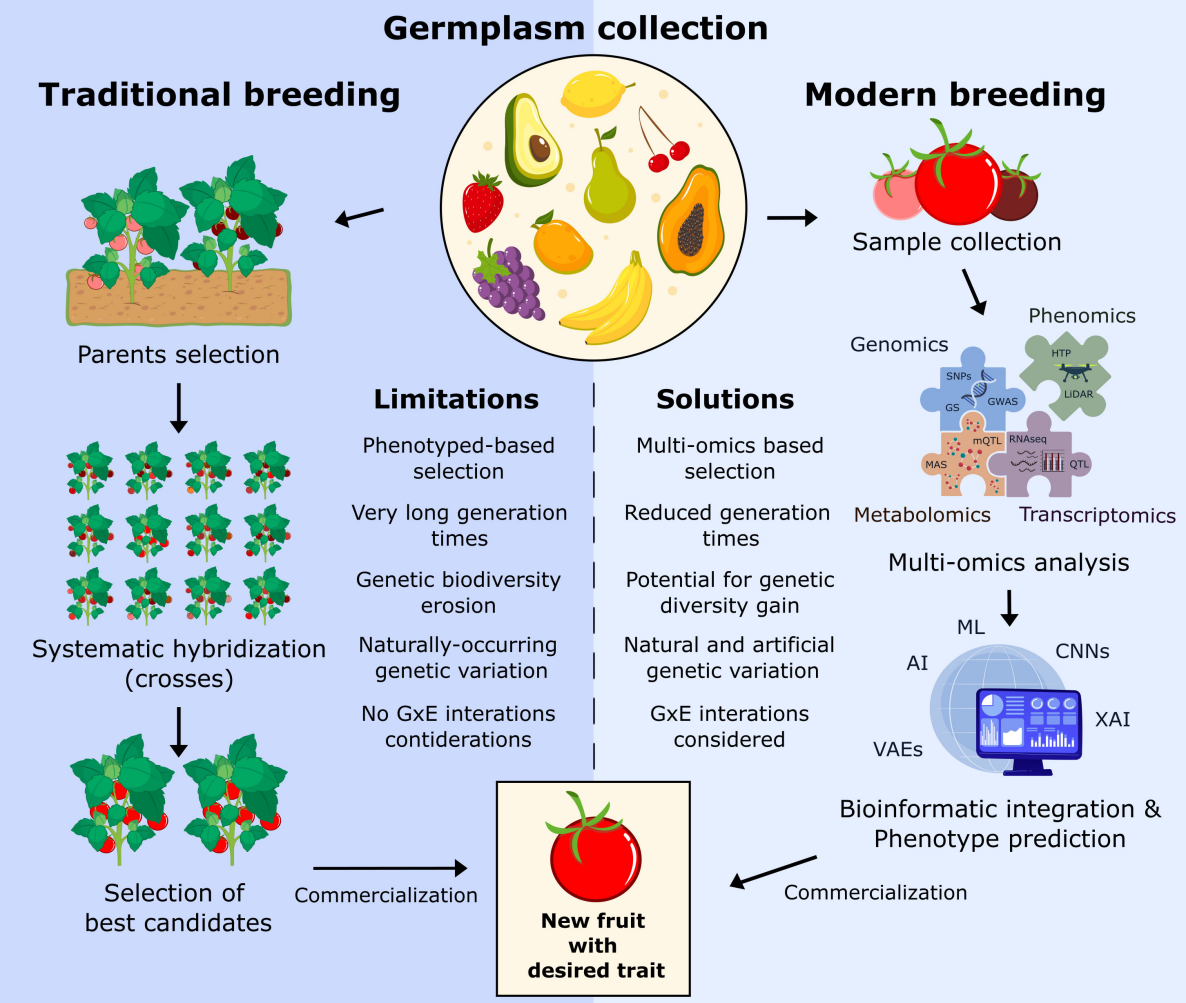
Traditional vs. modern breeding strategies for fruit crop improvement. The diagram provides a comparative overview of two distinct breeding paradigms. Traditional breeding involves the selection of parent plants, followed by repeated rounds of systematic hybridization and selection based on visible traits, a process that is time-consuming and often leads to genetic diversity erosion. In contrast, modern breeding employs an integrated pipeline beginning with sample collection and progressing through multi-omics analysis (genomics, transcriptomics, metabolomics, phenomics). Subsequent bioinformatic integration and AI-driven prediction enable the rapid identification and selection of desirable traits, ultimately leading to the commercialization of new cultivars with improved characteristics.

Over the last decade, fruit crop improvement has been driven by a complex relationship between empirical breeding and molecular biotechnology. While both methods have shaped fruit genetics, their different approaches have created unique advantages and challenges. The fundamental differences between traditional and modern breeding approaches are illustrated in **Figure 1**, highlighting the paradigm shift from phenotype-based selection to data-driven design.

### 2.1 Traditional breeding foundations: the genomics era

Plant breeding, one of humanity’s oldest agricultural practices, has shaped crops for human benefit from Neolithic selection to modern systematic hybridization (Acquaah, 2015; Bradshaw, 2017). Yet traditional methods proved inefficient (slow progress, phenotype dependence, GxE vulnerability) for fruit crops with long juvenile phases and complex genetics (Branchereau et al., 2023; Khan and Korban, 2022). Selection for commercial traits narrowed diversity, particularly pronounced in major fruit crops, including tomato and strawberry, where prioritizing elite genotypes has significantly curtailed the overall resilience of the germplasm pool (Aharoni et al., 2004; Khoury et al., 2022).

The genomic era, initiated with DNA structure elucidation and advancing through recombinant DNA to large-scale sequencing, provided unprecedented tools for understanding plant characteristics at fundamental levels (Cohen et al., 1973; Liu et al., 2023). This shift from phenotype-driven to genotype-driven design offers opportunities to recover diversity, shorten cycles, and access previously intractable traits (Kumar et al., 2014).

### 2.2 Marker-assisted selection in fruit crops

MAS is a modern breeding technique that exploits molecular markers (e.g. DNA variants) linked to target agronomic traits within segregating populations, enabling seedling-stage selection of individuals of interest, bypassing years waiting for phenotypic expression (De Mori and Cipriani, 2023). In apple breeding, MAS has successfully helped in the introgression of scab resistance genes within the commercially relevant cultivar “Fuji” (Rasool et al., 2024). This approach is commonly applied in combination with Genome-wide association studies (GWAS), which enable the identification of marker–trait associations directly within diverse germplasm collections, bypassing the need to develop segregating populations and shortening even more the generation times (Bauchet et al., 2017). However, the mapping resolution of GWAS is critically dependent on the extent and pattern of LD within each population, which can vary widely among germplasm panels (Z. Li and Zhou, 2025). The combination of the two techniques has proven to be effective to achieve breeding objectives in many relevant fruit crops, improving the efficiency of the process (**Table 1**). However, MAS excels for simply inherited traits but struggles with polygenic characteristics like flavor and resilience—exposing gaps between genomic resources and predictive capacity for consumer-relevant traits (Hagenguth et al., 2024; Ru et al., 2015).

**Table 1:** Overview of marker-assisted selection (MAS) and genomic selection (GS) applications in major fruit crops, showing population parameters (linkage disequilibrium, kinship coefficients) and prediction accuracies.

Species	Trait	N° Population	Marker	Accuracy	Reported LD o kinship	Reference
Strawberry (*Fragaria × ananassa*)	Fruit weight, shape uniformity, achene position, skin color, etc.	223 accessions	Identification of around 25 markers across several genomic regions	71 associations identified with 11 quality traits; markers explained between 10% of the phenotypic variance.	High Linkage Disequilibrium (LD) observed in some regions; Kinship matrix (K matrix) used in Mixed Linear Model (MLM) to account for population structure.	Prohaska et al., 2024
Sweet cherry (*Prunus avium*)	Fruit size	247 individuals	4.919 SNP markers identified across two populations	Genomic prediction accuracy ranged from 0.38 to 0.53 depending on population and year	Sub-populations analyzed individually and combined; LD spanning from 0,75 to 0,11. Kinship values reported.	McCord et al., 2024
Apple (Malus × domestica)	Red skin color intensity	160 accessions	SSR marker Mdo.chr9.4 (linked to MdMYB1)	Mdo.chr9.4-R0 explains 52% of phenotypic variance.	Kinship was employed for control of familial relatedness. The authors declared the LD estimation was performed as described by Moriya et al., 2017.	Moriya et al., 2017
Peach (*Prunus persica *L. Batsch)	Stony hard (SH) fruit texture	87 accessions	SSR marker (TC microsatellite in PpYUC11-like intron)	Perfect co-segregation of the PpYUC11-like intron TC20 allele and the SH phenotype validated by bi-parental F2 populations.	The authors measured intra-chromosomal LD patterns, restricting the *hd* locus to 1.8 Mbp; they also suggested possible "strong local LD around the SH locus".	Cirilli et al., 2018
Strawberry (*Fragaria x ananassa*)	Petiole length, leaf area, Brix, fruit hardness, pericarp color	105 inbred parental lines; 275 F1 hybrids for model training; 5,460 F1 hybrids predicted	SNPs (20,811 detected by whole-genome sequencing)	Including dominance effects slightly improved prediction accuracy; models showed predictability for most traits except Brix (specific values not reported)	Cross-population prediction between parents and F1 hybrids. LD maximum set to 0.95. No Kinship values reported.	Yamamoto et al., 2021
Oil palm (*Elaeis guineensis*)	7 oil yield components (e.g., bunch production, oil content)	~500 hybrid crosses (training), ~200 crosses (validation)	>5,000 SNPs (GBS); Genomic BLUP (GBLUP)	Prediction accuracy for cross values: 0.28–0.73 (trait-dependent); >10% gain in bunch production with GS preselection	Relationship matrix; Kinship not reported; LD not reported.	Cros et al., 2017
Avocado (*Persea americana*)	17 morpho-agronomic traits (seed weight, fruit weight, etc.)	104 elite “criollo” trees; 22 half-sib seedling families used as rootstocks in a 5-year common garden trial	13 SSRs; GBLUP	Narrow-sense heritability (h²) in elite “criollo” trees: 0.28–0.51 (max. 0.51 for adaxial veins); h² for agronomic traits: 0.34–0.39; in grafted Hass: h²=0.36 (number of fruits), h²=0.11 (fruit weight)	Relationship matrix; Kinship not reported; LD not reported.	Cañas-Gutiérrez et al., 2022
Cacao (*Theobroma cacao *L.)	Cadmium (Cd) uptake, growth, physiology rootstock × scion × soil interaction	320 progenies	Pedigree-based best linear unbiased prediction (ABLUP)	h² > 0.1 for several traits before grafting; h² < 0.1 after grafting or in soils without Cd	Pedigree-based relatedness matrix; heat plots used for verification. No Kinship or LD not reported.	Fernández-Paz et al., 2021
Coffe (*Coffea arabica*)	18 agronomic traits (e.g., yield, disease resistance, plant architecture)	195 individuals	21,211 SNPs; GBLUP	Moderate to high heritability and selective accuracy for most traits; GS can reduce cycle time by 50% (exact values not reported)	Kinship: Genomic relationship matrix (G-matrix); LD not reported.	Sousa et al., 2019
Avocado (*Persea americana*)	Fruit skin color, shape and taste	110 accessions	Identification of 12 markers within 11 genomic regions	Markers explained between 14.84% and 43.96% of the phenotypic variance, with an average of 24.63%.	LD estimation across three populations (Guatemalan, West Indian, Mexican). Kinship was employed to address population stratification in the GWAS analyses.	Li et al., 2024

### 2.3 Genomic selection: predictive power for complex traits

GS leverages genome-wide SNPs to predict breeding values of unphenotyped individuals, offering radical timeline compression (Bharati et al., 2023; Kostick et al., 2023). This technology is known to offer higher sensitivity while better controlling for linkage disequilibrium (LD), therefore surpassing pedigree-based alternatives (Daetwyler et al., 2012). Applications in strawberry improved firmness and color (Yamamoto et al., 2021) (**Table 1**), while peach achieved moderate predictions for quality traits (Gogorcena et al., 2020). Yet predictive power depends critically on reference population quality and phenotypic resolution—requirements that fruit crops’ complex structures and profound GxE interactions stretch to limits (Crossa et al., 2017; Pour-Aboughadareh et al., 2022). Additionally, few GS studies in fruit crops systematically report critical parameters such as LD distribution, precise kinship coefficients, or comprehensive cross-validation details (**Table 1**). This lack of standardized reporting significantly hinders the comparative evaluation of results and compromises the successful transferability of prediction models across diverse breeding populations.

### 2.4 Genetic transformation and gene editing

Transformation and editing enable direct genome modification, bypassing recombination constraints (Daniel et al., 2023). Transgenic papaya saved Hawaii’s industry (Gonsalves and Ferreira, 2003), while CRISPR achieved 500% lycopene increase through targeted editing (Zsögön et al., 2018). Despite breakthroughs, regulatory frameworks treat single-nucleotide edits like transgenics, creating decade-long approval delays. Long-term ecological consequences remain unresolved, risking genetic bottlenecks if editing focuses disproportionately on elite germplasm (Eckerstorfer et al., 2021; Halford, 2019).

### 2.5 Rootstock breeding: a specialized avenue for crop improvement

Rootstocks manage tree vigor, confer resistance to soil-borne pests like *Phylloxera*, and enable adaptation to abiotic stress, making them essential for sustainable fruit production (Gregory et al., 2013; Warschefsky et al., 2016). Specific breeding objectives for these subterranean partners focus on traits like reduced tree size, precocity (early fruiting), increased disease resistance, and enhanced nutrient and water use efficiency (Bowman et al., 2021; Rasool et al., 2020; Vahdati et al., 2021). This specialized avenue allows breeders to decouple and target the enhancement of above- and below-ground traits for improved fruit yield and quality (Ling et al., 2025). The last decade has seen a critical shift in accelerating these breeding cycles through advanced genomic and biotechnological tools. Genomic Selection (GS) is now being deployed to boost the predictive accuracy of complex traits like vigor control and stress tolerance, circumventing the long juvenile periods characteristic of perennial systems (E. J. Warschefsky et al., 2016). Recent quantitative trait loci (QTL) mapping studies have identified key genetic regions for root traits in grapevine and a three-locus model for dwarfing in apple, facilitating the use of Marker-Assisted Selection (MAS) to expedite new variety development (Bassett et al., 2015; Blois et al., 2023; Harrison et al., 2016). CRISPR-Cas enables precise editing of genes underlying rootstock-scion interactions, transforming the development of climate-resilient rootstocks. However, commercial deployment remains constrained by regulatory frameworks and validation timelines (Nishitani et al., 2016).

Despite the extensive publications in fruit rootstock breeding, translation to commercial breeding remains limited. Complex heterozygosity, protracted juvenile phases, and genotype-by-environment dependencies continue to impede the predictive accuracy of genomic models in perennial systems (Warschefsky et al., 2016). The lack of integrative multi-omics databases and the cost of long-term field validation further constrain industrial scalability. Thus, rootstock breeding—though a promising frontier for resilient fruit systems (Gregory et al., 2013)—requires further efforts to realize its full potential.

### 2.6 Impact on fruit crop biodiversity: a comparison of conventional breeding and modern approaches

Conventional fruit breeding delivered yield and uniformity at severe genetic cost, eroding adaptability and flavor (Luo et al., 2022; Williams and Clair, 1993). Modern tools offer potential reversal: MAS reintroduces beneficial alleles efficiently (Jiménez et al., 2025), while CRISPR enables precise modifications without disrupting genetic identity (Ali, 2024; Bohra et al., 2022). Yet concentration on elite lines and commercial traits risks reinforcing homogenization unless deliberately coupled with biodiversity-centered frameworks utilizing wild relatives and underrepresented germplasm (Marappan et al., 2025; Salgotra and Chauhan, 2023).

## 3 Integrating high-throughput non-destructive phenotyping for enhanced breeding outcomes: post-genomics era

The molecular revolution has given fruit breeders unprecedented tools to manipulate genomes, but progress continues to be throttled by an old obstacle: phenotyping (Song et al., 2021). While we can identify beneficial alleles in days and edit genomes in weeks, measuring the complex traits that truly matter (flavor, resilience, postharvest quality) remains slow, destructive, and often unreliable. This persistent “phenotyping bottleneck” (Nguyen et al., 2025) continues to stall the translation of genomic potential into cultivars.

Non-destructive high-throughput phenotyping (HTP) platforms are redefining this landscape through imaging, spectroscopy, and sensor-based approaches that allow repeated, real-time monitoring across the life cycle without compromising sample integrity (Anjali et al., 2024; J. Liu et al., 2025). These systems generate continuous, high-dimensional datasets that scale phenotyping efforts and reveal trait dynamics over time (Lou et al., 2015). When coupled with machine learning, they create frameworks for connecting molecular variation to agronomic performance (Solovchenko et al., 2023). **Table 1** summarizes the key HTP technologies, their applications, and current limitations.

### 3.1 Image-based high-throughput phenomics

Image-based technologies have redefined fruit phenotyping by enabling rapid, scalable measurement of morphological, physiological, and biochemical traits (Abebe et al., 2023).

#### 3.1.1 RGB imaging

RGB imaging enables automated assessment of fruit morphological traits including color, shape, and surface defects. Systems like FruitPhenoBox demonstrate throughput of thousands of fruits daily for genome-wide association studies (Kirchgessner et al., 2024). In apple, RGB imaging has been employed in combination to metabolomics to dissect the phenotypical and biochemical complexities of flesh pigmentation (Bouillon et al., 2024), a very complex trait of high commercial interest.

However, RGB captures only visible surface changes without revealing underlying biochemical mechanisms—a limitation addressed by complementary technologies. (See **Supplementary Text S1** for technical specifications).

#### 3.1.2 Hyperspectral imaging

Hyperspectral imaging (HSI) captures data across hundreds of spectral bands (400–2500 nm), enabling simultaneous evaluation of morphological and biochemical parameters (Li et al., 2023). This technology assesses internal quality traits (ripeness, sugar content, diseases) before visible symptoms appear, transforming phenotyping from descriptive to predictive (Sarić et al., 2022). In tomato, RGB-based HTP was able to effectively differentiate between the effects of biotic from abiotic stress and resistant/tolerant from susceptible genotypes representing a great tool to apply timely and appropriate actions (Prigigallo et al., 2025).

Current challenges include high costs, data volumes, and model transferability across populations. (Technical details in **Supplementary Text S1**).

#### 3.1.3 Thermal imaging

Thermal imaging measures infrared radiation to assess physiological status through temperature variations linked to water status, transpiration, and metabolic activity (Vadivambal and Jayas, 2011). In this study (Gonzalez-Dugo et al., 2013), an unmanned aerial vehicle equipped with a thermal imaging camera was employed to enhance and automate irrigation management in a commercial orchard comprising five fruit tree species, demonstrating the significant potential of these technologies for precision agriculture. The technology detects stress responses and internal disorders days before visible symptoms, critical for resilience breeding (Pathmanaban et al., 2022; Wen et al., 2023). However, environmental sensitivity remains a challenge. (See **Supplementary Text S1** for protocols).

#### 3.1.4 Fluorescence imaging

Fluorescence imaging quantifies photosynthetic efficiency by measuring chlorophyll re-emission patterns under specific light excitation (Abebe et al., 2023). This reveals plant stress responses and recovery capacity through parameters like Fv/Fm ratios, enabling early selection of stress-tolerant genotypes (Hashim et al., 2020; Momin et al., 2023). (Measurement parameters detailed in **Supplementary Text S1**.) For example, this technology has been integrated with high-performance liquid chromatography (HPLC) analysis to strawberry fruits to facilitate the screening of anthocyanin-rich genotypes (Yoshioka et al., 2013).

#### 3.1.5 3D modeling and tomography imaging

Three-dimensional reconstruction and tomographic approaches capture volumetric fruit structure through X-ray CT, MRI, or structured light scanning (Wang et al., 2024). These techniques measure internal architecture (seed development, cavities, tissue organization) with remarkable accuracy, valuable for breeding uniform morphology (Lu et al., 2023; Tuomainen et al., 2024). Nondestructive 3D phenotyping has been already applied to fruit with success. In particular, X-ray micro-computed tomography (micro-CT) has been used to automatically quantify fourteen morphological traits in passion fruit, facilitating the analysis of genotype–phenotype relationships aimed at breeding higher-quality cultivars (Lu et al., 2023). (Technical implementations in **Supplementary Text S1**).

### 3.2 Spectroscopy-based high-throughput phenomics

Spectroscopy analyzes light-matter interactions to decode metabolic states non-destructively (da Silva Alves et al., 2025), providing biochemical fingerprints complementary to imaging approaches.

#### 3.2.1 Visible-near infrared spectroscopy

Vis-NIR spectroscopy (400–2500 nm) rapidly translates spectral signals into predictions of sugars, acids, water, and specialized metabolites (Fernández-Novales et al., 2019). Strong correlations with destructive measurements offer cost-effective quality assessment (Amoriello et al., 2025; Grabska et al., 2023). For instance, Vis–NIR and portable Vis–NIR spectrophotometers were employed to differentiate apricot cultivars and build multi-cultivar, multi-year models for marketable quality traits using machine learning. This nondestructive approach proved an effective alternative to conventional methods for assessing fruit quality in both field and postharvest conditions (Amoriello et al., 2025). However, calibration models remain crop- and environment-specific, limiting broader application. (Calibration protocols in **Supplementary Text S1**).

#### 3.2.2 Dual-channel co-spectroscopy

Dual-channel systems merge reflectance and transmittance measurements, providing enhanced depth profiling of internal composition (Liang et al., 2025). This approach improves prediction robustness compared to single-channel systems, particularly for heterogeneous fruits. Recently, it was validated as a cost-effective and accurate method which employed artificial neural networks (ANN) for nondestructive assessment of soluble solids content in the commercial ‘Fuji’ apple cultivar (Liang et al., 2025). However, implementation costs and complexity currently limit widespread adoption. (System configurations detailed in **Supplementary Text S1**).

### 3.3 Other emerging non-invasive technologies

#### 3.3.1 Acoustics and vibrometry

Acoustic analysis measures fruit firmness through elastic wave propagation, linking texture to molecular processes like pectin degradation (Contador et al., 2015). QTL identification for firmness-related alleles enables marker-assisted selection, shortening breeding cycles (Di Guardo et al., 2017). Translation across environments remains challenging given the polygenic nature of texture traits. (Technical specifications in **Supplementary Text S1**).

#### 3.3.2 Robotics and automation

Robotic platforms enable field-scale phenotyping through integration of sensors on mobile systems (Oliveira et al., 2021). UAVs capture orchard-level data rapidly, while ground robots perform detailed plant-specific analysis (Fonteijn et al., 2021; Li et al., 2025). This multi-scale approach accelerates selection while providing temporal dynamics throughout growing seasons (Trybała et al., 2024; Xia et al., 2025). (Platform specifications in **Supplementary Text S1**).

**Table 2** summarizes the key HTP technologies currently available, their measurement principles, applications in fruit breeding, and critical limitations that must be addressed.

**Table 2:** Summary of high-throughput phenotyping technologies for fruit breeding.

Technology	Primary measurement principle	Key quality traits assessed	Link to omics (genomics/metabolomics)	Key advantage	Research gap/limitation
RGB Imaging	Detection of reflected visible light (red, green, blue bands, 380–760 nm)	Color, size, shape, growth dynamics, superficial disorders	Provides large-scale phenomic datasets to integrate with SNP genotyping for GWAS and genomic prediction	Low-cost, highly accessible, scalable to thousands of samples	Limited to surface traits; lacks biochemical/physiological insight
Hyperspectral Imaging (HSI)	Acquisition of hundreds of narrow, continuous spectral bands	Ripeness, sugar/acid content, internal defects, stress and disease signatures	Links metabolic signatures to genetic loci, bridging genotype–phenotype gaps	Mechanistic, non-destructive, early stress detection	High cost, complex data processing, limited field scalability
Visible-Near Infrared (Vis-NIR) Spectroscopy	Absorption and scattering of light by chemical bonds (O-H, C-H)	Soluble Solids Content (SSC), firmness, acidity, sugar/acid ratio	Provides a proxy for the metabolome to enhance genomic selection (G) models	Non-destructive, rapid, and cost-effective; can serve as a phenomic proxy for genotyping	Model robustness across cultivars and environments; lacks direct mechanistic link
Dual-Channel Co-Spectroscopy	Simultaneous measurement of transmitted and reflected light signals	SSC, internal quality, external defects	Generates a richer, more holistic dataset for predictive modeling of complex traits	Captures both internal and external information in a single measurement	Novelty; lack of extensive validation across species; scalability
Acoustics and Vibrometry	Mechanical response to sound or vibration	Firmness, texture, maturity	Directly measures the physical phenotype linked to cell wall modifications, allowing for the identification of texture-related QTLs	Objective, direct measurement of a critical trait for consumer appeal	Primarily focused on a single trait; sensitivity to fruit geometry; mechanistic links require further omics integration
Robotics and Automation (UAVs, conveyors, robotic arms)	Automated multi-sensor data acquisition platforms	Multi-trait phenotyping (morphology, physiology, stress, yield)	Enables high-throughput integration of phenomics with genomics/metabolomics at population scale	Increases throughput, reduces labor cost, standardizes phenotyping	Limited adoption in field-scale fruit crops; high initial investment

Technologies are categorized by measurement principle, target traits, breeding applications, advantages, and current limitations that constrain widespread adoption.

### 3.4 Challenges and limitations of high-throughput phenotyping in fruit breeding

Despite transformative potential, HTP faces critical limitations. Equipment costs ($50,000-$250,000 for hyperspectral systems) restrict adoption to well-funded programs (Yang et al., 2020). Inconsistent calibration protocols and environmental variability undermine data comparability across experiments (Kim, 2020). Most critically, the data deluge strains computational infrastructure while machine learning models struggle with transferability across species and environments (M. Zhang et al., 2023). High-throughput phenotyping has revolutionized data collection, yet we now face a paradox: drowning in high-dimensional datasets while lacking frameworks to extract biological meaning. Traditional statistics collapse under non-linear interactions and multi-omics complexity. As explored next, machine learning promises to decode these hidden relationships—though bringing its own limitations of black-box predictions and uncomfortable dependence on data quality.

## 4 Machine learning algorithms applied to multi-omics integrative approaches in fruit breeding strategies

Multi-omics integration represents fruit breeding’s most pressing challenge and opportunity. The incorporation of omic-based strategies has demonstrated significant potential to advance sustainability, quality, and resilience goals in the near future. In the context of rootstock breeding, a widely used tool for perennial fruit species, transcriptomic approaches combined with Weighted Gene Co-expression Network Analysis (WGCNA) revealed in grapevine that the scion genotype modulates the rootstock’s transcriptomic response to low phosphate conditions (Gautier et al., 2020). Gene modules and potential hub genes associated with phosphate treatment and scion/rootstock genotypes were identified, providing evidence of putative signaling interactions. In another grapevine study, metabolomics identified markers predictive of grafting success as early as 33 days post-grafting, markedly preceding traditional evaluation methods (Loupit et al., 2022). These approaches typically rely on single-omic analyses and basic statistical methods, limiting their capacity to resolve biological complexity. Multi-omics frameworks extract substantially more information. In strawberry, integrating genomic, transcriptomic, and volatile metabolic profiles precisely identified flavor-associated genes and their regulatory elements through GWAS, subsequently validated by transient gene expression analyses (Fan et al., 2022). Similarly, in peach, the combination of genomics, transcriptomics, and metabolomics elucidated a chemical roadmap for fruit aroma by identifying volatiles linked to consumer preference. Notably, the study uncovered a trade-off in which red-fleshed accessions exhibited substantial reductions in key flavor volatiles such as linalool. The NAC transcription factor PpBL was identified as a central regulator within a gene regulatory network (GRN05) controlling both color and volatile profiles, while haplotypes of three tandem PpAAT genes were strongly associated with decreased ester content (Cao et al., 2024). These findings offer valuable insights for fruit breeding programs, deepening our understanding of complex trait regulation and paving the way for their integration into commercial breeding strategies.

Despite progress enabled by multi-omics approaches, traditional statistical methods often fail to capture the non-linear, high-dimensional relationships inherent in genomic, metabolomic, and phenomic datasets, leaving much of the underlying biological variation unexplored. Machine learning (ML) emerges as the bridge, enabling predictive modeling and pattern extraction from biological complexity (van Dijk et al., 2021; Ferrão et al., 2023). Classical correlation methods overlook epistatic interactions and metabolic regulation. ML algorithms (random forests, SVMs, gradient boosting, ANN) accommodate non-linearities and feature interactions, demonstrating superior genomic selection performance (Aasim et al., 2023; Ergün, 2025; Gu et al., 2025; Yeon et al., 2024). These approaches integrate thousands of SNPs with metabolomic and phenotypic data for trait prediction (Fan et al., 2025; Ferrão et al., 2021), though requiring large, annotated datasets scarce in fruit breeding. All these new technologies are already being implemented in combination with omic approaches to elucidate new pathways for sustainable fruit breeding and production (**Table 3**).

**Table 3:** Representative multi-omics integration studies in fruit crops.

Fruit crop	Target trait	Multi-Omic layers	Major findings	Reference
Tomato (*Solanum lycopersicum*)	Fruit quality traits (Brix, lycopene content, colour, etc)	Phenomics (refractometric analysis, spectrophotometric analysis), Machine learning (XGBoost Model, ANN Model, SHAP value analysis)	A robust comparative analysis leveraging Phenomics and Machine Learning demonstrated the superior predictive capacity of the XGBoost model over ANN for key tomato fruit quality parameters. XGBoost achieved exceptionally high accuracy for °Brix (R^2^ = 0.98) and strong performance for lycopene (R^2^ = 0.87) and color ratio (R^2^ = 0.93). Critically, the ANN model's substantial failure in color prediction (R^2^ = -0.35) highlights the necessity of rigorous model selection in predictive breeding applications, despite the utility of SHAP value analysis in confirming feature importance across both models for Brix and lycopene.	M’hamdi et al., 2024
Lemon (*Citrus limon*)	Disease (HLB) response, early detection	Transcriptomics (RNA-Seq), Proteomics (MS/MS), Metabolomics (H NMR)	The multi-omics integration successfully delineated the early lemon HLB caused by *Candidatus Liberibacter asiaticus*. This multi-pronged approach provided critical insights for the identification of presymptomatic biomarkers, establishing a strong molecular foundation for the development of highly sensitive, early diagnostic technologies crucial for effective disease management and quarantine efforts in citrus production.	Ramsey et al., 2020
Colombian Caribbean avocados and Hass	Bioactive compound diversity and metabolomic profile	Metabolomics (comparative untargeted metabolomic analysis)	Results revealed significant metabolomic diversity between Colombian Caribbean and Hass avocados, identifying unique bioactive compounds and metabolic signatures relevant for nutritional quality and potential breeding targets. Findings assessed the chemical uniqueness of the cultivars and highlighted their potential for food and industrial applications.	Rodriguez et al., 2025
Ruby Red grapefruit (*Citrus × paradisi* Macf.)	Diplodia stem-end rot caused by *Lasiodiplodia theobromae*	Metabolomics (UHPLC/MS, GC/MS), Machine learning (GBT, RF, MLP, SVM, logistic regression)	This Metabolomics-Machine Learning pipeline demonstrated the power of early postharvest disease detection. The authors were able to diagnose *Lasiodiplodia theobromae* infection in grapefruit one full week before visual symptoms manifested, based on distinct metabolic profiles. The identification of plant hormone signaling, phenylpropanoid biosynthesis, and glutamate metabolism as primary defense pathways provides critical, targeted avenues for developing preventative postharvest treatments.	An et al., 2024
Passion fruit (*Passiflora edulis*)	Fruit color and aroma	Genomics (genome assembly and annotation), Transcriptomics (RNA-Seq), Metabolomics (UPLC-MS/MS)	Integrative multi-omics profiling of purple and yellow passion fruit revealed extensive genomic variation, including millions of SNPs and large presence/absence variations. Joint analysis of genomic, transcriptomic, and metabolomic data identified candidate genes and metabolic pathways—especially those involved in flavonoid and anthocyanin biosynthesis—underlying differences in fruit color. Terpenoid metabolites were found to accumulate more in purple passion fruit, contributing to aroma differences. Functional characterization of terpene synthase genes demonstrated both shared and unique terpene products between varieties, providing a genetic and metabolic basis for the distinct aroma and flavor profiles. These findings offer valuable resources for genetic improvement and breeding of passion fruit.	Zheng et al., 2024
Grape (*Vitis vinifera*)	Berry size, postharvest quality, decay, shriveling, weight loss	Genomics (GWAS), Phenomics (refractometric analysis)	A GWAS study across 588 grape cultivars successfully pinpointed novel QTLs governing both pre- and post-harvest quality. Researchers identified key QTL for berry fruit width linked to Vitvi11g000454, suggesting a critical role for jasmonic acid signaling in berry development. This work is highly valuable as it provides immediately actionable candidate genes and molecular markers to accelerate breeding programs aimed at improving berry size and reducing postharvest decay/shriveling via MAS.	García-Abadillo et al., 2024
Loquat (*Eriobotrya japonica*)	Fruit weight	Genomics (QTL mapping), Transcriptomics (RNA-seq), Metabolomics (LC–MS/MS)	The authors identified three major loci associated with fruit weight. Auxin metabolism was found to play a key role during fruit enlargement. Candidate genes (EjEIN4, EjTRN1) and SNP markers were identified, providing new insights and molecular tools for breeding loquat with improved fruit weight.	Peng et al., 2022
Peach (*Prunus persica *L. Batsch)	fruit shape, fruit flesh adhesion, soluble solids concentration, titratable acidity, and flesh Color	Genomics (Whole-genome sequencing), Phenomics (Traditional), Machine Learning model based on linear regression	The authors constructed genetic maps using resequencing data to enhance the QTL analysis mapping resolution. They developed a Machine Learning (ML)-based model to assess flesh color, which proved more efficient than physical colorimetric parameters, detecting consistent QTLs. Finally, they established positions for two potential candidate genes and nine QTLs for quantitative qualily traits, which could be used for further studies in relation to peach fruit quality breeding.	Fan et al., 2025

Examples demonstrate how combining genomic, transcriptomic, metabolomic and machine learning approaches reveals complex trait regulation and rootstock-scion interactions that single-omics analyses miss.

Deep learning marks a paradigm shift through direct learning from high-dimensional data. CNNs and RNNs excel in image phenotyping and multi-omics integration (Minamikawa et al., 2022; F. Yang et al., 2024). Generative AI opens new frontiers—GANs augment limited datasets while VAEs simulate metabolic landscapes, enabling hypothesis testing before costly trials (Bird et al., 2022; Gomari et al., 2022). Graph neural networks model biological networks, improving predictions while generating mechanistic hypotheses (Han et al., 2024).

Despite predictive power, deep networks remain “black boxes” with limited interpretability. Explainable AI approaches, such as SHAP, integrated gradients and attention mechanisms, identify contributing markers and pathways (Chowdhury et al., 2024; Novielli et al., 2024). Critical challenges persist: data imbalance, limited samples, overfitting, and lack of standardized pipelines complicate reproducibility (Atkins et al., 2025; Zarbakhsh et al., 2025). Solutions require collaborative consortia, open repositories, and hybrid models combining mechanistic biology with data inference.

## 5 Current research gaps, controversies, and future perspectives of integrative approaches

Multi-omics integration promises to decode complex traits and accelerate breeding, yet persistent gaps limit practical applications beyond proof-of-concept studies (R. Zhang et al., 2022). The challenge has shifted from technical viability to examining whether innovations can deliver without compromising equity, transparency, or genetic diversity.

Integration faces computational hurdles: heterogeneous datasets require complex normalization while batch effects and missing data hinder robust pipelines like mixOmics and MOFA+ (Arancibia-Guerra et al., 2025; Argelaguet et al., 2018). Terabyte-scale datasets create infrastructure paradoxes where algorithm development depends on data difficult to generate and share (Siddique et al., 2025).

Critical gaps persist in causal inference: correlations abound but causal regulators remain elusive (Zingaretti et al., 2020). Models successful for simple traits fail when applied to polygenic, environment-sensitive characteristics. Poor transferability across populations undermines the central justification for omics approaches in diversity utilization (E. Warschefsky et al., 2014). Machine learning compounds interpretability challenges through black-box predictions that reduce breeder confidence.

Logistical barriers limit adoption: sequencing costs, computing infrastructure, and interdisciplinary expertise requirements concentrate capabilities in well-funded consortia, raising equity concerns (Lassoued et al., 2021; Siepel, 2019). Integration with metabolomics remains rudimentary—genomic associations lack biochemical context for flavor, nutrition, and resilience traits exhibiting high plasticity (Gong et al., 2024).

Future success depends less on technological breakthroughs than on frameworks balancing innovation with accessibility, causality with complexity, and commercial demands with conservation. Advances in spatial omics and affordable phenotyping may address technical hurdles, but equally critical are collaborative infrastructures ensuring benefits extend beyond elite programs to diverse traits, crops, and regions. Only by addressing these gaps can integrative omics fulfill promises such as both innovation drivers and diversity safeguards, making this tension the field’s defining challenge.

## 6 Beyond the data: the new bottlenecks in the agri-tech revolution

The promises of integrative omics and advanced breeding are often presented as linear success stories—gene discovery, genome editing, trait validation, cultivar release. Yet the reality is far more fragmented. The path from “computer to field” and from “field to consumer” is obstructed by bottlenecks that data alone cannot solve. Failures are not due to weak science, but to the biological, regulatory, socioeconomic, and phenotypic barriers that still define agriculture.

**Figure 2:**
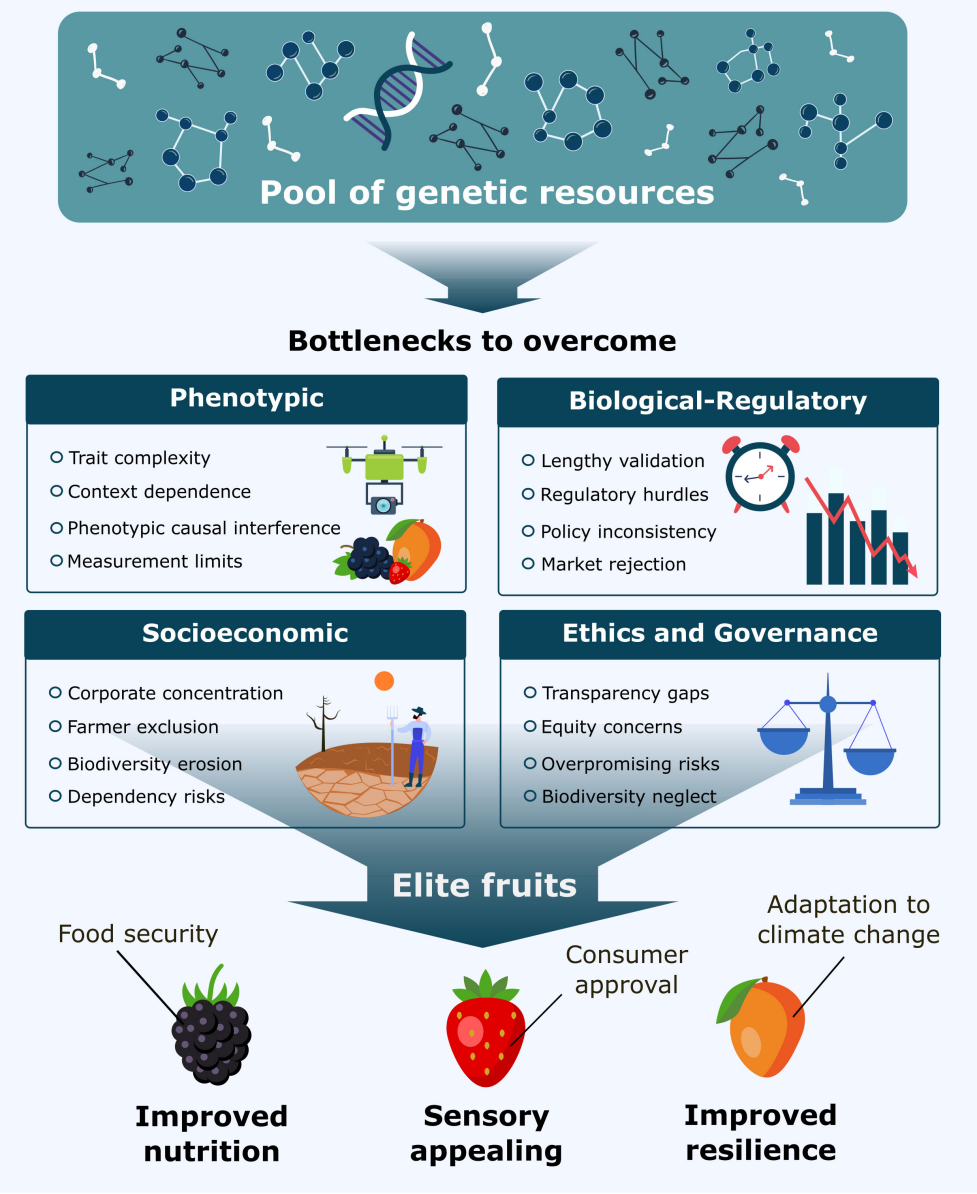
Conceptual framework illustrating key bottlenecks in modern fruit crop improvement. Genetic resources form the foundation for breeding, but their translation into elite fruits is constrained by phenotypic, biological-regulatory, socioeconomic, and governance barriers. Overcoming these challenges is essential to deliver improved nutrition, sensory quality, and resilience, ensuring food security, consumer approval, and adaptation to climate change.

The biological-regulatory bottleneck exposes a fundamental paradox in modern breeding. We can now engineer a 500% increase in tomato lycopene content in six months (Zsögön et al., 2018), yet the same tomato may spend more than 10 years and €14,5 million navigating Europe’s regulatory mazes (Smyth et al., 2017)—if it ever reaches market at all. Consider this: CRISPR-edited crops with single nucleotide changes identical to natural mutations require the same regulatory scrutiny as transgenic organisms carrying bacterial genes. The results? While Chinese consumers already eat CRISPR-edited tomatoes enriched with GABA for blood pressure control (Waltz, 2021), European farmers still wait for approval of blight-resistant potatoes that could significantly reduce fungicide use (Namanda et al., 2004). Technology has advanced exponentially; the regulatory framework remains frozen in 1990s logic. These interconnected bottlenecks form a complex landscape that constrains the translation of genomic discoveries into agricultural reality (**Figure 2**).

Similarly, the socioeconomic bottleneck reveals an uncomfortable truth: the ‘democratization of data’ narrative is a myth. Four corporations now control 66% of global seed sales (Canfield et al., 2021), up from 29% in 1996 - and every major genomic breeding platform requires partnerships with these same players. A basic HTP phenotyping setup costs 500,000 euros, while the computational infrastructure for genomic selection demands another 200,000 euros annually (Kim, 2020; Yang et al., 2020).

The result is a vicious cycle: only large corporations can afford the technology which they use to develop varieties that further consolidate their market position. Meanwhile, the more than 500 million smallholder farmers who produce 80% of the world’s food in developing countries (Lowder et al., 2021) are not just excluded from the genomic revolution - they’re becoming more dependent on its corporate gatekeepers. Is this technological progress or digital colonialism with a genomic face?

Lastly, the ‘last mile’ phenotypic bottleneck mocks our technological hubris: we’ve identified 40+ QTLs controlling tomato flavor (Yang et al., 2025), sequenced thousands of accessions, and can predict fruit size with 90% accuracy (Wang B. et al., 2024) - yet consumer complaints about tasteless tomatoes have increased exponentially since 2000. Why? Because the traits that matter most - the burst of umami when you bite a tomato, the lingering sweetness of a strawberry, the way a peach’s aroma changes 48 hours post-harvest - involve volatile metabolites so context-dependent that our million-euro phenotyping platforms only capture a small percentage of the variance. The bitter irony: a trained sensory panel of five humans still outperforms our best metabolomic predictions for consumer preference. We’re drowning in data about traits consumers ignore while remaining blind to qualities they value.

Therefore, as future perspectives, we encourage researchers to conduct studies aimed at identifying and quantifying the factors that contribute to the existing ‘Agri-Tech Valley of Death’ that difficult the effective translation of biotechnological discoveries into industrial applications across different areas of fruit improvement. To this end, future compilations might benefit from PRISMA systematic searches and SWOT frameworks for balanced technology assessment to evaluate the impact of these limitations, uncover novel factors that may negatively influence the transfer of knowledge to industry, and ultimately develop preventive or corrective strategies that help overcome these barriers and achieve sustainable production of high-quality fruits. A series of strategic recommendations for advancing future fruit breeding programs is presented in **Box 1**. We anticipate that the adoption of these strategies will significantly accelerate the achievement of key sustainability and resilience goals.

Box 1 **Strategic recommendations for accelerating fruit breeding programs. Actions target the biological-regulatory, socioeconomic, phenotypic, and governance bottlenecks that prevent genomic discoveries from reaching farmers and consumers.**

**Table:** 

Current Bottleneck	Strategic Recommendation	Key Stakeholders
I. Data Heterogeneity and Incomparability	Develop and adopt standardized phenotyping protocols for all high-throughput equipment and core traits (e.g., firmness, sugar/acid balance). This will ensure data calibration and comparability across different research laboratories and breeding sites.	Academic consortia, Equipment manufacturers, Public and Private Breeding Programs
II. Insufficient Training Data for AI/ML	Establish public, benchmark multi-omics datasets—including genomic, transcriptomic, and dense phenomic data—to train and rigorously validate Machine Learning (ML) and Genomic Prediction (GP) models.	Funding Agencies, International Genome Initiatives (e.g., Rosaceae), Public Research Institutions
III. Limited Model Transferability	Implement flexible transfer learning frameworks that enable the adaptation of predictive models (e.g., trained in one species or environment) to related fruit crops or different environmental conditions.	Bioinformatics specialists, Computational Breeding Programs
IV. Lack of Genomic Selection Reporting Standards	Develop and enforce rigorous reporting guidelines for GS studies that mandate the inclusion of key parameters: Linkage Disequilibrium (LD) distribution, precise Kinship coefficients, training/testing population ratios, and various model prediction accuracies.	Scientific Journals, Reviewers, Global Plant Breeding Societies
V. Slow Genetic Gain due to Long Breeding Cycles	Establish pre-competitive consortia to facilitate the secure and rapid exchange of genomic and phenomic data between public and pre-competitive private breeding programs to maximize Training Population (TP) size and power.	Public Research Institutes, Pre-competitive Private Sector, Government Agencies
VI. Regulatory Hurdles for Gene Editing	Establish rapid, risk-based regulatory pathways for genome-edited products that do not introduce foreign DNA (e.g., cis-gene editing). This will expedite the deployment of high-precision GS and gene-editing outputs.	Government Regulatory Bodies, Policy Makers, Agricultural Industry
VII. Loss of Genetic Diversity	Integrate genetic diversity conservation as an explicit, quantifiable metric within all breeding goals. GS must not only maximize short-term gain but also manage long-term genetic variance.	Breeders, Geneticists, Policy Makers

Against this background, we can continue celebrating technological acceleration while ignoring that farmers still plant decades-old varieties and consumers still complain about quality. Or we can acknowledge an uncomfortable truth: the bottleneck was never really about data or technology. It was always about biology’s irreducible complexity, regulation’s necessary caution, and human preferences that no algorithm fully captures. The path forward requires not more data, but more wisdom about which data matters. Not faster computers, but better questions. Not just genomic prediction, but genuine dialogue between breeders, farmers, and consumers. The real paradigm shift won’t come from our machines - it will come from admitting their limitations.
