# Supplementary material for: From data to decisions: a paradigm shift in fruit agriculture through the integration of multi-omics, modern phenotyping, and cutting-edge bioinformatic tools
Source: Front Plant Sci. 2025 Dec 10;16:1707289. doi: 10.3389/fpls.2025.1707289 (PMC12727975; doi:10.3389/fpls.2025.1707289)
Supplement: Supplementary file 1 [file DataSheet1.docx]

**Supplementary Text S1: Technical Details of High-Throughput Phenotyping Technologies in Fruit Breeding**

**S1. Image-Based High-Throughput Phenotyping Technologies**

**S1.1 RGB Imaging - Technical Specifications**

RGB imaging operates on the principle of capturing visible light (400-700 nm wavelength) reflected from fruit surfaces using standard charge-coupled device (CCD) or complementary metal-oxide-semiconductor (CMOS) sensors. Modern systems achieve resolutions up to 50 megapixels with color depth of 24-bit (16.7 million colors), enabling detection of subtle color variations imperceptible to human observers.

The FruitPhenoBox system exemplifies state-of-the-art implementation, incorporating six synchronized cameras capturing fruits from multiple angles at 120° intervals. This configuration generates point clouds of approximately 2 million vertices per fruit, enabling reconstruction accuracy of ±0.5 mm for size measurements and ±2° for color space coordinates (Kirchgessner et al., 2024).

However, RGB imaging faces inherent limitations in penetration depth (surface-only analysis), sensitivity to illumination variations (requiring controlled lighting environments with coefficient of variation <5%), and inability to detect sub-surface defects or internal quality parameters. Shadow artifacts and specular reflections on waxy fruit surfaces can introduce measurement errors of 10-15% if not properly controlled through polarizing filters and diffuse illumination systems.

**S1.2 Hyperspectral Imaging - Detailed Mechanisms**

Hyperspectral imaging (HSI) extends beyond visible spectrum analysis by capturing electromagnetic radiation across 200-400 contiguous spectral bands spanning UV (200 nm) through near-infrared (2500 nm) wavelengths. Each pixel contains a complete spectrum, generating data cubes of dimensions X × Y × λ where spatial resolution typically ranges from 1024×1024 pixels and spectral resolution achieves 2-10 nm full width at half maximum (FWHM).

The technology employs various scanning modalities: pushbroom scanners for conveyor systems (processing 100 fruits/minute), wavelength scanning for stationary samples (5-10 seconds per fruit), and snapshot imaging for rapid assessment (30 fps). Chemometric models using partial least squares regression (PLSR) or support vector machines achieve R² values of 0.85-0.95 for soluble solids content, 0.80-0.90 for acidity, and 0.75-0.85 for firmness predictions (Li et al., 2023).

Critical limitations include high equipment costs ($50,000-$250,000), substantial data volumes (2-10 GB per fruit for full spectral cubes), computational requirements for real-time processing (requiring GPU acceleration for throughput >10 fruits/minute), and model transferability challenges across cultivars and growing seasons (requiring annual recalibration with n>100 samples).

**S1.3 Thermal Imaging - Physiological Assessment**

Thermal cameras detect infrared radiation (7.5-14 μm wavelength) emitted from fruit surfaces, converting temperature variations into electrical signals via microbolometer arrays or cooled photon detectors. Modern uncooled sensors achieve temperature resolution of 0.02-0.05°C with spatial resolution up to 1024×768 pixels and frame rates of 30-60 Hz for dynamic process monitoring.

The Stefan-Boltzmann law governs emission intensity (E = εσT⁴), where emissivity (ε) varies among fruit surfaces (0.94-0.98 for most fruits). Temperature gradients correlate with transpiration rates (r = 0.82), metabolic activity (r = 0.76), and internal disorders (detection accuracy 78-92% for watercore in apples). Early disease detection achieves 85-95% accuracy 3-5 days before visible symptoms through identification of 0.5-2°C temperature anomalies at infection sites (Baranowski et al., 2012).

Environmental factors significantly influence measurements: ambient temperature fluctuations >2°C/hour reduce accuracy by 30%, relative humidity variations affect evaporative cooling patterns, and air currents >0.5 m/s introduce convective heat transfer artifacts. Standardized protocols require 20-30 minute equilibration periods and controlled environment chambers maintaining ±0.5°C and ±5% RH stability.

**S1.4 Fluorescence Imaging - Photosynthetic Efficiency**

Chlorophyll fluorescence imaging quantifies photosystem II quantum efficiency through pulse-amplitude-modulated (PAM) fluorometry. Excitation with blue (450-495 nm) or red (620-655 nm) light induces chlorophyll-a emission at 680-740 nm. The Kautsky effect generates characteristic fluorescence transients (OJIP curve) over 1-second timescales, encoding information about photosynthetic apparatus status.

Key parameters include: F₀ (minimal fluorescence in dark-adapted state), Fm (maximal fluorescence), Fv/Fm ratio (maximum quantum efficiency, healthy range 0.78-0.84), Y(II) (effective quantum yield), NPQ (non-photochemical quenching), and qP (photochemical quenching). Stress detection sensitivity reaches 0.05 Fv/Fm units, identifying dysfunction 7-14 days before visible symptoms with 89% accuracy (Momin et al., 2023).

Technical requirements include dark adaptation periods (15-30 minutes), precise irradiance control (0-3000 μmol photons m⁻² s⁻¹), and temperature stabilization (±1°C) as fluorescence exhibits -0.002 Fv/Fm units per °C temperature coefficient. Measurement protocols vary among species requiring optimization of saturation pulse intensity (3000-8000 μmol m⁻² s⁻¹) and actinic light levels.

**S1.5 3D Modeling and Tomographic Imaging**

Three-dimensional reconstruction employs multiple technologies: structured light scanning (accuracy ±0.1 mm), photogrammetry (50-200 images per fruit), time-of-flight cameras (30 fps, ±5 mm accuracy), and laser triangulation (sampling density 500 points/cm²). Advanced algorithms including Poisson surface reconstruction and ball pivoting achieve watertight meshes with 100,000-500,000 triangular faces per fruit.

X-ray computed tomography provides internal structure visualization at 20-200 μm voxel resolution. Micro-CT systems operating at 40-225 kV generate 600-2400 projections over 360° rotation, reconstructed via filtered back-projection or iterative algorithms. Contrast resolution of 0.5% enables differentiation of tissues varying by 10-20 Hounsfield units. Applications include seed counting (98% accuracy), internal disorder detection (92% sensitivity), and tissue density mapping (r² = 0.91 with destructive measurements).

Magnetic resonance imaging offers complementary soft tissue contrast through T1 (500-2000 ms) and T2 (50-150 ms) relaxation time mapping at 0.2-1.5 Tesla field strengths. Water content quantification achieves ±2% accuracy, while metabolite imaging via ¹H-MRS detects sugars at 10-50 mM concentrations. Scan times range from 2-30 minutes limiting throughput to 20-50 fruits/day.
