# Supplementary material for: From data to decisions: a paradigm shift in fruit agriculture through the integration of multi-omics, modern phenotyping, and cutting-edge bioinformatic tools
Source: Front Plant Sci. 2025 Dec 10;16:1707289. doi: 10.3389/fpls.2025.1707289 (PMC12727975; doi:10.3389/fpls.2025.1707289)
Supplement: Supplementary file 2 [file DataSheet2.docx]

**Supplementary Text S2: Spectroscopy-Based Phenotyping Technologies**

**S2.1 Visible-Near Infrared (Vis-NIR) Spectroscopy - Molecular Fingerprinting**

Vis-NIR spectroscopy (400-2500 nm) exploits molecular overtones and combination bands arising from C-H, O-H, N-H, and C=O bond vibrations. Specific absorption features correlate with quality parameters: water (970, 1450, 1940 nm), sugars (910, 1440, 2320 nm), acids (1680-1750 nm), and pigments (420-550 nm for carotenoids, 520-700 nm for anthocyanins).

Instrumentation configurations include dispersive spectrometers (0.5-2 nm resolution), Fourier transform systems (0.02 nm resolution), and linear variable filter designs for portable applications. Integration times range from milliseconds (LED sources) to seconds (halogen lamps) with signal-to-noise ratios exceeding 10,000:1 for research-grade instruments. Fiber optic probes enable flexible sampling geometries: diffuse reflectance (0°/45°), transmittance, and interactance modes optimized for different fruit sizes and optical properties.

Multivariate calibration models require extensive datasets (n>200 samples spanning harvest seasons) with wet chemistry reference analyses. Model performance metrics include: root mean square error of prediction (RMSEP) of 0.3-0.8 °Brix for SSC, 0.05-0.15% for titratable acidity, 2-5 N for firmness, with ratio of performance to deviation (RPD) values >2.5 indicating quantitative prediction capability.

**S2.2 Dual-Channel Co-Spectroscopy - Enhanced Penetration Depth**

Dual-channel systems simultaneously acquire reflectance and transmittance spectra, providing complementary information from surface (0-2 mm) and bulk (5-30 mm) tissues. Spatial offset configurations position source and detector fibers 0-30 mm apart, enabling depth-resolved measurements through photon time-of-flight discrimination.

The modified Beer-Lambert law incorporating scattering effects (μs' = 0.5-2 mm⁻¹ for fruit tissues) governs light propagation: I = I₀exp(-μaL)S(μs',L) where absorption coefficient μa ranges 0.001-0.1 mm⁻¹. Monte Carlo simulations optimize source-detector geometries maximizing sampling volume while maintaining adequate signal (>10³ photons/nm). Multi-distance measurements (5, 10, 15, 20 mm) enable spatially-resolved spectroscopy extracting depth-specific composition profiles.

System complexity increases with additional components: bifurcated fiber bundles ($2,000-5,000), dual spectrometers ($15,000-30,000), and specialized light sources (supercontinuum lasers $50,000+). However, prediction accuracy improvements of 15-25% for heterogeneous fruits justify investments in high-value crop applications.
