# Supplementary material for: From data to decisions: a paradigm shift in fruit agriculture through the integration of multi-omics, modern phenotyping, and cutting-edge bioinformatic tools
Source: Front Plant Sci. 2025 Dec 10;16:1707289. doi: 10.3389/fpls.2025.1707289 (PMC12727975; doi:10.3389/fpls.2025.1707289)
Supplement: Supplementary file 3 [file DataSheet3.docx]

**Supplementary Text S3: Emerging Non-Invasive Technologies**

**S3.1 Acoustic and Vibrometry Analysis**

Acoustic firmness sensors operate on principles of elastic wave propagation through viscoelastic fruit tissues. Impact excitation (0.1-1 N force) generates acoustic emissions (100 Hz - 20 kHz) detected by accelerometers or laser Doppler vibrometers. The dominant frequency (f₀) relates to firmness through: f₀² = (E/ρ) × (geometric factor) where E represents elastic modulus (0.1-10 MPa) and ρ density (800-1050 kg/m³).

Frequency resolution of 0.1 Hz enables detection of 0.5 N firmness changes. Temperature compensation (+2.5 Hz/°C) and size normalization (f₀ × m^0.33) improve correlation with destructive measurements (r = 0.85-0.95). Machine learning models incorporating 10-15 spectral features achieve classification accuracy of 92-96% for harvest maturity determination.

Advanced implementations employ continuous wave excitation (swept sine 50-5000 Hz) measuring transfer functions. Modal analysis identifies resonance modes corresponding to tissue layers: skin (2-5 kHz), flesh (200-800 Hz), and core (50-200 Hz). Damping ratios (0.02-0.15) indicate internal defects with 88% sensitivity. Non-contact laser vibrometry eliminates loading effects achieving repeatability <2% coefficient of variation.

**S3.2 Robotic Platforms and Field Deployment**

Agricultural robotics integrate multiple sensing modalities on mobile platforms navigating orchards at 0.5-2 m/s. Simultaneous localization and mapping (SLAM) algorithms achieve ±5 cm positioning accuracy using RTK-GPS, LiDAR (±2 cm at 100 m range), and visual odometry. Computer vision systems employing convolutional neural networks (YOLOv5, Mask R-CNN) detect fruits with 85-95% accuracy under variable illumination (100-100,000 lux).

Manipulator specifications include 6-7 degrees of freedom, ±0.5 mm repeatability, 2-5 kg payload capacity, and compliant gripping (0.5-5 N force control). Harvesting success rates reach 75-90% at 3-8 seconds per fruit depending on canopy density and occlusion levels. Multi-spectral cameras (RGB-D, NIR, thermal) mounted on end-effectors enable quality assessment during harvesting with onboard processing via edge computing devices (NVIDIA Jetson, Intel NUC).

Unmanned aerial vehicles equipped with gimbal-stabilized sensors capture orchard-scale data at 2-10 cm/pixel ground resolution. Flight parameters (altitude 10-50 m, speed 2-8 m/s, overlap 70-85%) optimize coverage versus battery life (15-45 minutes). Photogrammetric processing generates orthomosaics and digital surface models enabling individual tree segmentation, canopy volume estimation (R² = 0.89), and yield prediction (±15% accuracy) weeks before harvest.
