# Supplementary material for: From data to decisions: a paradigm shift in fruit agriculture through the integration of multi-omics, modern phenotyping, and cutting-edge bioinformatic tools
Source: Front Plant Sci. 2025 Dec 10;16:1707289. doi: 10.3389/fpls.2025.1707289 (PMC12727975; doi:10.3389/fpls.2025.1707289)
Supplement: Supplementary file 4 [file DataSheet4.docx]

**Supplementary Text S4: Integration Challenges and Solutions**

**S4.1 Data Management Infrastructure**

Multi-modal phenotyping generates heterogeneous datasets: images (10-500 MB/fruit), spectra (1-10 MB), 3D models (50-200 MB), and time series (1-100 MB). Annual breeding programs evaluating 10,000 genotypes × 50 fruits × 5 technologies produce 10-50 TB requiring hierarchical storage systems: SSD for active processing (10 TB, $3,000), NAS for recent data (100 TB, $15,000), and cloud/tape archives for long-term storage ($0.01-0.02/GB/month).

Database architectures employ NoSQL solutions (MongoDB, Cassandra) for unstructured data and relational systems (PostgreSQL) for metadata. Standardized schemas following MIAPPE (Minimum Information About Plant Phenotyping Experiment) guidelines ensure interoperability. RESTful APIs enable programmatic access with authentication via OAuth 2.0 and rate limiting (1000 requests/hour) preventing system overload.

**S4.2 Computational Requirements**

Real-time processing demands parallel computing architectures: multi-core CPUs (16-64 cores) for general tasks, GPUs (NVIDIA RTX 3090, A100) accelerating deep learning inference (10-100× speedup), and FPGAs for embedded systems. Distributed computing frameworks (Apache Spark, Dask) enable horizontal scaling across clusters.

Algorithm optimization strategies include: image pyramid processing reducing computation by 75%, region-of-interest extraction decreasing data volume by 90%, and model quantization (INT8) achieving 4× inference speedup with <2% accuracy loss. Edge computing on sensor platforms performs initial filtering reducing network traffic by 80-95%.

**S4.3 Calibration and Standardization**

Cross-platform calibration requires reference standards: color charts (X-Rite ColorChecker, ΔE < 2), dimensional artifacts (±0.01 mm certified spheres), and chemical standards (certified reference materials ±1% uncertainty). Transfer models using direct standardization or piecewise direct standardization maintain prediction accuracy (RMSEP increase <15%) across instruments.

Temporal drift monitoring via control charts (Shewhart, CUSUM) detects 2σ deviations triggering recalibration. Environmental compensation models incorporate temperature (±0.1°C), humidity (±2% RH), and atmospheric pressure (±1 hPa) maintaining measurement uncertainty <5% across operating conditions (5-40°C, 20-90% RH).
